# The role of emotional regulation on early child school adjustment outcomes

**DOI:** 10.1016/j.apnu.2024.07.003

**Published:** 2024-07-02

**Authors:** Harry Adynski, Cathi Propper, Linda Beeber, John H. Gilmore, Baiming Zou, Hudson P. Santos

**Affiliations:** a National Clinician Scholars Program, Philip R. Lee Institute for Health Policy Studies, University of California San Francisco, CA, United States; b School of Nursing, University of North Carolina at Chapel Hill, NC, United States; c Department of Psychiatry, University of North Carolina at Chapel Hill, NC, United States; d Department of Biostatistics, University of North Carolina at Chapel Hill, NC, United States; e The University of Miami School of Nursing and Health Studies, FL, United States

**Keywords:** Early childhood, Child development, Emotional regulation, Psychiatric nursing, School adjustment, Mental health promotion

## Abstract

Emotional regulation involves managing attention, affect, and behavior, and is essential for long-term health and well-being, including positive school adjustment. The purpose of this secondary data analysis from the Durham Child Health and Development Study was to explore how parent and teacher reported emotional regulation behaviors related to school adjustment outcomes (social skills, academic performance, and academic achievement) during early childhood. Parent and teacher reports on emotional regulation behaviors showed mixed concordance, however they correlated with critical aspects of school adjustment. Clinical and practical implications are discussed, including the role of psychiatric nurses in promoting positive emotional regulation and school adjustment outcomes across settings.

## Introduction

Emotional regulation includes an individuals' ability to manage attention, affect, and behavior to meet goals (Thompson, 2019). Emotional regulation directly affects child development, health, and wellbeing, and sets the path for an individual to manage health behaviors and meet their full social and economic potential across their lifespan. One in four pre-school aged children from low-income families experience poor emotional regulation (hereafter emotional dysregulation) (Brown et al., 2012). Childhood emotional dysregulation develops due to the complex interplay of individual, family, community, and societal factors, which all affect a child's susceptibility. Developmentally, emotional regulation rapidly changes from infancy to early childhood as there is a marked shift from innate reactivity or reflexive behaviors and parental coregulation (McIntosh et al., 2021) to more purposeful and independent means of regulating (Calkins & Marcovitch, 2010). During early childhood, children begin to integrate emerging cognitive and behavioral strategies to meet personal goals and social demands (Blankson et al., 2017). Emotional dysregulation has been identified as a potential mechanism contributing to adverse physical and mental health across the lifespan (Althoff, Verhulst, et al., 2010; Beauchaine & Cicchetti, 2019; Cavicchioli et al., 2022; Ros & Graziano, 2020).

In addition to its influence on long term health outcomes, emotional regulation sets the foundation for successful school adjustment with the acquisition of basic socioemotional and academic competencies (Blair & Raver, 2015; Eisenberg et al., 2010; Noronã-Zhou & Tung, 2020). As children enter school, they are challenged to adjust to new social and academic demands, including interacting with teachers, caregivers, and classmates. Emotional regulation is an essential element of school adjustment which encompasses the interaction between an individual child's social, emotional, physical, and intellectual development and their environment including the school, family, and community resources that support the developing child (Head Start, 2021; Johnson, 2017; Williams & Lerner, 2019). In the United States (U.S.), there is increasing evidence of widening gaps in aspects of school adjustment, including disparities in social-emotional development and early academic skills (Peterson et al., 2018). For example, children growing up in poverty show lower levels of self-control (similar to emotional regulation) compared to their age-matched peers (Reardon & Portilla, 2016). Differences in emotional regulation may be a mechanism that drives school adjustment disparities, including social skills (Calkins & Marcovitch, 2010; Dollar et al., 2022; Howard et al., 2022; Robson et al., 2020), academic performance (Li et al., 2017), and academic achievement (math and literacy skills) (Edossa et al., 2018; McClelland et al., 2007; Morrison et al., 2010; Sawyer et al., 2015; Skibbe et al., 2019). Additionally, emotional regulation has been predictive of long-term education attainment, including completion of high school and college attendance (Duncan et al., 2010; Johnson et al., 2022) and long-term employment (Robson et al., 2020). Child emotional regulation is a key child health outcome, and, given its implications for long-term well-being, should be targeted for intervention to help children reach their full social, vocational, and economic potentials across the lifespan (Waxmonsky et al., 2021).

School entry is often a critical time for nurses, caregivers, teachers, psychologists, and/or counselors to screen and identify children who are having difficulties with emotional regulation and academic adjustment (Holochwost et al., 2021). Caregivers and teachers are the most common informants on assessing childhood emotional regulation behaviors, however parent and teacher reports are often weakly associated (Aitken et al., 2019; Rescorla et al., 2018). Despite these weak associations, the use of multiple informants is essential to disentangle whether a child's emotional regulation behaviors are based on stable characteristics (trait i.e. temperament or genetic) or are dependent on specific situations or contexts (state i.e. home, caregiver, or school environment) (Carducci & Nave, 2020). Children who exhibit high reactivity (trait) may show increased vulnerability to developing emotional dysregulation. While sensitive parenting at home can help mitigate this tendency, navigating the school environment may pose greater challenges and stressors for a child to effectively regulate their emotions. The distinction between state and trait characteristics of emotional regulation allows for a better understanding of whether emotional regulation behaviors change over time and across settings. For example, a parent may observe high emotional regulation at home, while a teacher may observe low emotional regulation in the classroom. A child may experience increased stress and anxiety at school for a variety of reasons including social or academic challenges, while these regulatory challenges may not be present at home indicating a child's difficulty with state rather than trait-based factors. Understanding how children express emotional regulation behaviors across informants and contexts can facilitate appropriate screening and intervention efforts. This informs whether early emotional regulation behaviors matter for later school adjustment and whether they should be targeted before school entry based on trait characteristics. This can inform the implementation of parenting, early care, and education-based interventions to reduce child socioemotional problems and challenges with school adjustment (Duncan et al., 2018; Harrington et al., 2020).

This paper is guided by the Social Ecological Model, which posits that health and disease are influenced by multiple factors across the individual, organizational, community and societal levels and that these factors interact in various ways to predict health outcomes (McLeroy et al., 1988). The conceptual model developed by Harrington et al. (2020) offers a framework that may explain the proposed relationships between emotional regulation and academic and socioemotional components of school readiness. For example, individual-level factors that are both related to child emotional regulation and school adjustment include children's sex assigned at birth or child temperament (Klassen & Stewart, 2022; Mitchison et al., 2020; Williams et al., 2016; Williams & Howard, 2020). Family level factors include maternal education (Asmussen et al., 2022), marital status (Cross, 2020), and income level (Duncan et al., 2010; Hetherington et al., 2020; Kao et al., 2020; Miller et al., 2021). Societal and systemic factors related to income disparities and structural racism which marginalize certain racial and ethnic groups also contribute to disparities in school outcomes (Henry et al., 2020; Iruka et al., 2022; Zulauf-McCurdy & Loomis, 2022). These factors interact across multiple levels and contribute to a child's later emotional regulation and school adjustment.

In the current study, we utilize secondary data from the Durham Child Health and Development Study to explore the state/trait characteristics of emotional regulation behaviors (from home [parent] and school [teacher]) and subsequent school adjustment outcomes (teacher reported academic performance and social skills) as well as standardized academic achievement assessments accounting for individual and family level characteristics. The following research questions were developed: 1) Do child emotional regulation behaviors correlate across home (parent report) and school (teacher report) settings? 2) Do parent-reported emotional regulation behaviors during early childhood correlate with school adjustment outcomes (social skills & academic performance, academic achievement) at kindergarten, first, and second grade? 3) Do teacher reported emotional regulation behaviors at school entry correlate with future academic achievement at second grade? Understanding how parent and teacher reports on emotional regulation relate to school adjustment outcomes can guide the timing of screening, parenting, early care, and education-based interventions. This knowledge can also aid psychiatric nurses in promoting lifelong health and wellbeing across the lifespan.

## Methods

The present study utilizes data from the Durham Child Health and Development Study and was approved by the University of North Carolina at Chapel Hill institutional review board (#21–0231). This community based longitudinal cohort study began in 2002. The parent study investigated early child development in mother-child dyads through multiple in-home, laboratory, and mail-in school questionnaires from infancy into early childhood. Despite the age of the data, its value remains significant for several reasons including the use of developmentally appropriate measures of emotional regulation from multiple informants spanning from early childhood through the second grade. This dataset allows us to explore the relationship between emotional regulation and key school adjustment outcomes.

### Participants

All families from the original cohort were recruited from Durham North Carolina, a medium-sized city in the Southeast U.S. Families were recruited via fliers and postings at birth and parenting classes as well as through phone contact. Infants who were premature, had serious postnatal health complications or chromosomal, genetic, metabolic, or congenital abnormalities or defects evident at birth identified via birth records were excluded from the study. Parents consented to have study staff contact their child's teachers at kindergarten, first, and second grade. Teachers then completed consent forms and questionnaires via mail.

### Study timeline

The original data was collected in two waves. Wave 1 included parent assessments at 3, 6, 12, 18, 24, 30, and 36 months of age. Wave 2 included assessments at 60 (kindergarten), 72 (first grade), and 84 (second grade) months of age. Within the present study data was utilized from both waves of the original study starting at the 18-month visit. Demographic and parent reported emotional regulation measures were collected from wave 1 and teacher reported emotional regulation and school adjustment measures were collected from wave 2.

### Measures

#### Demographic and confounding variables

Demographic data was extracted from the 18-month visit and included maternal, family, and child level demographics. For maternal demographics, age (years) and education level (<high school (HS), ≥HS (HS, some college, college degree)) were collected. Family level demographics included marital status (married, non-married) and income based on the federal poverty level Income-Needs-Ratio (below 200 % and ≥ 200 %). Child level demographics included race/ethnicity and sex assigned at birth. Within the present analyses demographic variables were included as confounding variables including the child's sex assigned at birth (Klassen & Stewart, 2022; Mitchison et al., 2020; Williams et al., 2016; Williams & Howard, 2020), parental self-reported race/ethnicity (Henry et al., 2020; Iruka et al., 2022; Zulauf-McCurdy & Loomis, 2022), maternal education (Asmussen et al., 2022), parental marital status (Cross, 2020), and family income level (Duncan et al., 2010; Hetherington et al., 2020; Kao et al., 2020; Miller et al., 2021). These demographic variables were included as confounders as they are related to both emotional regulation behaviors and school adjustment outcomes.

#### Primary independent variable: emotional regulation measures

Emotional regulation was the primary independent variable in our analyses. Both parents and teachers completed three assessments of emotional regulation, including emotional lability/negativity, regulation, and dysregulation.

##### Child Behavior Checklist (parent).

The Child Behavior Checklist 1½−5 (CBCL) is a nationally normed measurement tool and is one of the most widely used caregiver report checklists that assess generalized psychopathology including emotional and behavioral problems of preschool aged children 1 ½ to 5 years old (Achenbach, 2019; Achenbach & Rescorla, 2001; Rescorla, 2005). The CBCL has demonstrated strong reliability with reported internal consistency, test-retest reliability, and inter-rater agreement (Achenbach & Rescorla, 2001). The CBCL includes 99 items in which mothers were asked to rate their child's behavior over the past 2 months on a 3-point Likert scale (not true of the child, somewhat true, very true or often true) with higher scores indicating greater prevalence of that behavior. The CBCL was administered at four time points before school entry (18, 24, 30, 36, months). The CBCL Dysregulation Profile (CBCL-DP) is calculated by combining the t-scores of three syndrome scales (Biederman et al., 2009). These scales measure three aspects of emotional regulation including emotional (anxious/depressed), cognitive (attention problems), and behavioral (aggressive behavior). Within community samples a score greater than or equal to 180 is commonly used to denote dysregulation (Carlson et al., 2016; Kim et al., 2012; Meyer et al., 2009). Final scores were the average of the 18–36 month score if a parent completed at least one questionnaire (*n* = 187). Within our sample the internal reliability for the CBCL-DP subscales were Anxiety/Depression (α = 0.54–0.69), Attention Problems (α =0.66–0.73), and Aggressive Behaviors (α =0.86–0.89).

##### Emotional Regulation Checklist (teacher/parent).

The Emotional Regulation Checklist (ERC) is a 24-item teacher or parent reported measure that indicates how often a child displays affective behaviors (Shields & Cicchetti, 1997). The ERC has demonstrated strong internal consistency or reliability in previous studies (Shields & Cicchetti, 1997). Within the present study, parents completed the ERC at 60 months while teachers completed the ERC at kindergarten, first, and second grade (children with 1 or more completed ERC assessment at kindergarten, first, and second grade *n* = 129). Parents and teachers used a four-point Likert scale to indicate how often a child displayed affective behaviors (never, sometimes, often, almost always), with higher scores indicating more frequent displays of behavior. The ERC has two subscales: emotional regulation (ERC-R) and emotion lability/negativity (ERC-L/N). The emotional regulation subscale assesses child adaptive regulatory behaviors that include socially appropriate displays of emotion and empathy. The lability/negativity subscale assesses mood lability, lack of flexibility, dysregulated negative affect, and inappropriate affective displays. Final ERC teacher scores used in analyses were the average across the kindergarten, first, and second grade scores if a teacher had completed at least one questionnaire (n = 129). The internal consistency for the ERC parent scores was 0.62 (ERC-R) and 0.82 (ERC-L/N). The internal consistency for the ERC teacher scores for ERC-R was (0.58–0.67), while the internal consistency for the ERC-L/N was (0.87–0.90).

##### Teacher Report Form (teacher).

The Teacher Report Form (TRF) is a nationally normed assessment tool that assesses a broad range of child behavioral and emotional problems. The TRF has demonstrated strong reliability with reported internal consistency between 0.72 and 0.97 for the empirically based subscales (Achenbach & Rescorla, 2001). The TRF includes 112 items in which teachers are asked to determine how well an item describes a child within the past two months. Each item uses a three-point Likert scale (not true, somewhat true, very true) with higher scores indicating greater prevalence of that behavior. The TRF was administered at kindergarten, first, and second grade. The TRF Dysregulation Profile (TRF-DP) is calculated by combining the t-scores of three syndrome scales as described earlier in the CBCL-DP (Biederman et al., 2009). Within our sample, the internal reliability for the TRF-DP subscales were Anxiety/Depression (α = 0.69–0.84), Attention Problems (α =0.90–0.92), and Aggressive Behaviors (α =0.86–0.87). The final scores used for analysis were the average of scores from kindergarten, first, and second-grade if at least one teacher report was completed.

#### Outcome variables: school adjustment

School adjustment outcome variables included teacher-reported social skills, academic performance, and standardized achievement assessments.

##### Social skills: SSRS-Social Skills.

The Social Skills Rating System (SSRS) is a teacher reported assessment which includes 57 items that assess social, behavioral, and academic adjustment. The SSRS Social Skills subscale (SSRS-SS) was used to assess child social skills at kindergarten, first, and second grade. The SSRS-SS includes the first 30 items of the scale and asks teachers how often a student exhibits social behaviors (i.e., self-control, interpersonal skills, verbal assertion) on a 3-point Likert scale (never, sometimes, very often) during the past two months. Within our sample the SSRS-SS demonstrated strong internal reliability with (α =0.91–0.93) across kindergarten, first, and second grade assessments. Final scores used in analyses for the SSRS-SS were the average of the kindergarten, first, and second grade scores if there was at least one completed teacher report (*n* = 129).

##### Academic performance: Academic Performance Rating Scale.

The Academic Performance Rating Scale (APRS) is a 19-item scale that was developed to reflect teachers' perceptions of children's academic performance and abilities in classroom settings. Individual items assess work performance in various subject areas, academic success, behavioral control in academic situations, attention to assignments, as well as the frequency of staring episodes and social withdrawal. Items are rated on a 6-point Likert scale with higher scores indicating greater classroom academic performance. The APRS includes three subscales: academic success, academic productivity, and impulse control (DuPaul et al., 1991). The APRS was administered at kindergarten, first, and second grade. The academic productivity (APRS-AP) and academic success (APRS-AS) subscales were used in the present analyses. The internal reliability for the APRS-AP subscale was 0.91–0.93 and APRS-AS was 0.78–0.93 across the kindergarten, first, and second grade assessments. Final scores used in analyses for the APRS-AP and APRS-AS were the average of the kindergarten, first, and second grade scores if there was at least one completed teacher report (*n* = 125).

##### Academic performance: SSRS-Academic Competence Scale.

The SSRS Academic Competence Scale (SSRS-AC) was also used to assess teacher reported academic performance. The last 8 items of the SSRS are used to calculate the SSRS-AC. Teachers are asked to rate children in their classroom according to a 5-point Likert scale comparing the child to other children in their class (1 = lowest 10 %, 2 = next lowest 20 %, 3 = middle 40 %, 4 = next highest 20 %, 5 = highest 10 %) based on academic competence in mathematics and reading, motivation to learn, parental encouragement, intellectual functioning, and overall classroom behavior. The SSRS-AC demonstrated strong internal reliability within our sample (α =0.94–0.95) across kindergarten, first, and second grade. Final scores used in analyses for the SSRS-AC were the average of the kindergarten, first, and second grade scores if there was at least one completed teacher report (*n* = 128).

##### Academic achievement: Woodcock Johnson III.

The Woodcock-Johnson Tests of Achievement III (WJ-III) is a widely used nationally normed academic achievement assessment that measures early math and literacy skills (Woodcock et al., 2001). The two subtests including Letter-Word Identification and Applied Problems subtests were administered in kindergarten, first, and second grade. The Letter-Word Identification test assesses a child's prereading and reading skills through word identification. Children are asked to identify letters that appear in a word and pronounce the words correctly. The Applied Problems subtest measures a child's ability to understand and complete math problems. Children are presented verbally with a word math problem, and then must identify the procedure, and complete the correct calculation. Within both subtests, children answer questions of increasing difficulty until they reach a ceiling level with six incorrect answers in a row. Both the Letter-Word Identification (LWI) and the Applied Problems (AP) subtests have strong internal reliability among school aged children 0.98 and 0.92 respectively (Woodcock et al., 2001). Raw scores were converted to grade based standard scores for both the WJII-AP and WJIII-LWI using computerized scoring technology (Woodcock et al., 2001). Within the present analyses the WJIII-AP (*n* = 120) and WJIII-LWI (*n* = 121) were completed at second grade.

### Statistical analysis

Descriptive analyses were conducted for the demographic variables, parent and teacher reported emotional regulation measures, and school adjustment variables. To maximize sample size and mitigate the impact of inconsistent individual response rates across measures, we calculated average scores for repeated measures. For example, if a participant had one response across multiple time points, we carried that response forward, whereas if they had two or more, we calculated the average of those scores. This method allowed for the inclusion of all available data points and minimized the influence of individual response inconsistencies, thereby enhancing the robustness of our findings. To explore the association between parent and teacher reported emotional regulation measures (dysregulation CBCL-DP, TRF-DP, lability/negativity [ERC-L/N], regulation [ERC-R]), correlation analysis was performed. Statistical significance was assessed at the significance level of 0.05.

**Fig. 1.**
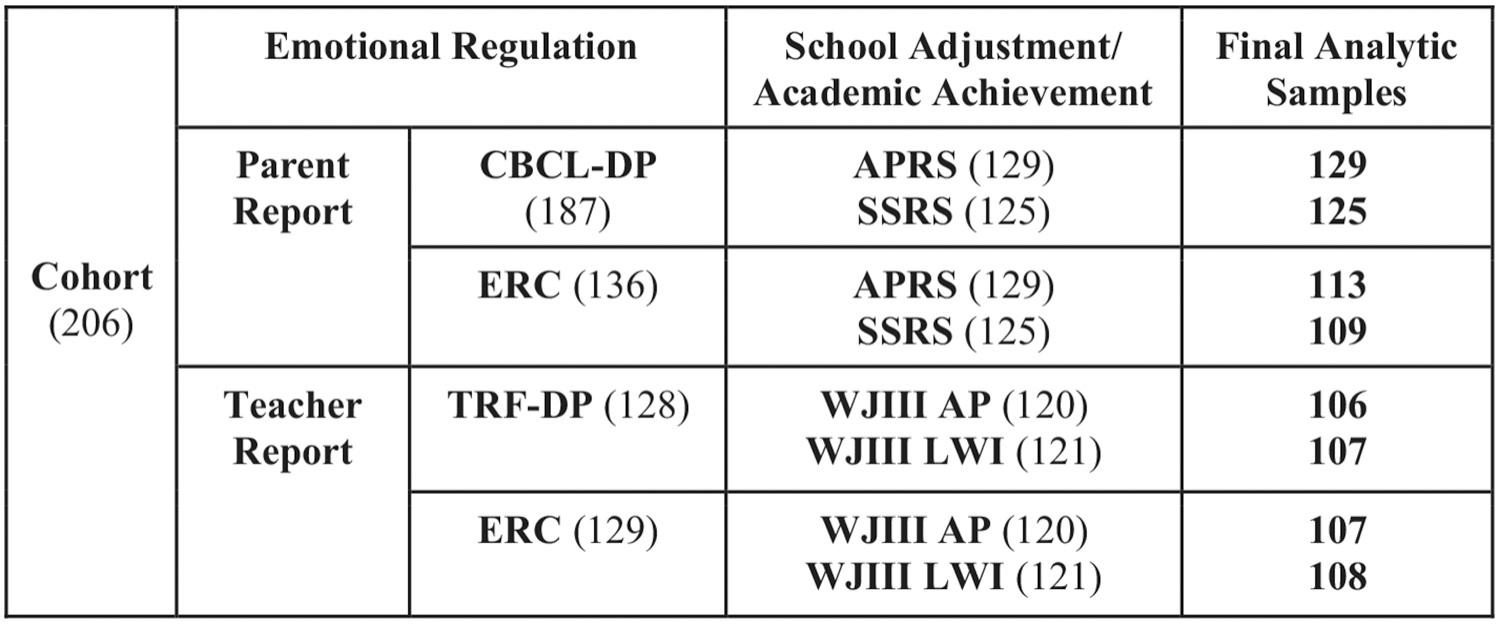
Participant flow chart for final analytic samples. Note: CBCL-DP = Child Behavior Checklist Dysregulation Profile (average score 18–36 months), TRF-DP = Teacher Report Form Dysregulation Profile (average score 60, 72, 84 month), ERC-L/N = Emotional Regulation Checklist Lability/Negativity (parent = 60 months, teacher = average score of 60, 72, 84 month), ERC-R Emotional Regulation Checklist Regulation (parent = 60 months, teacher = average score of 60, 72, 84 month), APRS (Academic Performance Rating Scale), SSRS (Social Skills Rating Scale), WJIII (Woodcock Johnson III) (AP = Applied Problems, LWI = Letter Word Identification). Final Analytic Sample sizes indicate the number of children who had a complete Emotional Regulation measure and outcome variable (Academic Adjustment/Academic Achievement).

In order to explore parent reported emotional regulation behaviors (CBCL-DP, ERC-L/N, ERC-R) effects on school adjustment outcomes, linear regression models were adopted with emotional regulation (CBCL-DP, ERC-L/N, or ERC-R) being set as the primary independent variable and school adjustment outcome as the dependent variable (SSRS or APRS). Linear regression models were adopted with the emotional regulation measure set as the primary independent variable and the academic achievement outcome as the dependent variable. To investigate the teacher-reported emotional regulation behaviors effects on standardized academic achievement scores at second grade linear regression models were adopted with emotional regulation (TRF-DP, ERC-L/N, ERC-R) set as the primary independent variable and academic achievement (WJIII AP or WJIII LWI) set as the dependent variable. We limited our analysis to explore the academic achievement outcomes at second grade to account for the temporality of the teacher reports across kindergarten, first, and second grade. The following confounding variables sex assigned at birth, race/ethnicity, maternal education, marital status, and income level were included within the analysis. For a summary of the final analytic sample sizes, see Fig. 1. Statistical significance was assessed at the significance level of 0.05. Parameter estimates, confidence intervals (CI), and *p*-values are reported for the linear regression models.

## Results

The sample had a mean maternal age of 29.3 (SD = 5.9) with a majority being married (62.6 %) and having a HS education or greater (87.9 %) (HS, some college, 4-year college degree). Families were roughly equal between the two income groups with a small majority being in the high-income group ≥200 % Income-Needs-Ratio (51.5 %). The sample consisted of 56 % Black or African American and 44 % White children, with sex assigned at birth being nearly evenly split between male (51.5 %) and female (48.5 %) children (see Table 1). See Table 2 for descriptive statistics for our primary predictors and outcome variables.

### Association between parent and teacher report of child emotional regulation

The parent reported emotional regulation score (CBCL-DP) was statistically significantly related to the accompanying parent-reported lability/negativity score (ERC-L/N) (ρ = 0.58, *p* ≤0.001) and regulation score (ERC-R) (ρ = 0.18, *p* = 0.025), but not statistically significantly related to any of the teacher reported measures of emotional regulation (TRF-DP, ERC-L/N, ERC-R). The teacher reported emotional dysregulation score (TRF-DP) was statistically significantly related to the accompanying teacher report lability/negativity score (ERC-L/N) (ρ = 0.79, *p* ≤0.001), and statistically significantly yet negatively associated with the regulation score (ERC-R) (ρ = −0.43, *p* ≤0.001). The parent reported lability/negativity score (ERC-L/N) was statistically significantly correlated to two of the three teacher reported regulation measures including the lability/negativity score (ERC-L/N) (ρ = 0.31, *p* = 0.001) and the emotional dysregulation score (TRF-DP) (ρ = 0.32, *p* = <001). The parent reported regulation score (ERC-R) was only statistically significantly related to the accompanying teacher reported regulation score (ERC-R) (ρ = 0.31, *p* = 0.001) (see Table 3).

### Parent report of child emotional regulation and academic adjustment (social skills, academic performance)

#### Social skills

Among the parent reported emotional regulation measures the lability/negativity (ERC-L/N) and emotional regulation (ERC-R) behaviors were statistically significant predictors of teacher reported social skills. For every one unit increase in ERC Lability/Negativity score there was a 0.40 (95 % CI [0.1, 0.6], *p* ≤0.001) decrease in social skills score. For every one unit increase of the ERC-regulation score there was a 0.57 (95 % CI [0.02, 1.12], *p* = 0.042) increase in SSRS-SS score (see Table 4).

#### Academic performance

The parent reported lability/negativity (ERC-L/N) behaviors were statistically significant and predicted all three academic performance outcomes including academic productivity (APRS-AP) (−0.5, 95 % CI [−0.7, −0.2], *p* = 0.002), academic success (APRS-AS) (−0.3, 95 % CI [−0.5, −0.1], *p* = 0.003), and academic competence (SSRS-AC) (−0.4, 95 % CI [−0.6, −0.2], *p* ≤0.001). The parent reported ERC-R subscale was a statistically significant predictor of only the academic performance outcome (APRS-AP) (0.75, 95 % CI [−0.23, −0.128], *p* = 0.005) (see Table 4).

### Teacher report of child emotional regulation and academic adjustment (academic achievement)

Linear regression was used to examine the relationship between teacher reported emotional regulation (TRF-DP, ERC- L/N, ERC-R) behaviors, and academic achievement (WJIII AP and WJII LWI) at second grade.

#### Academic achievement

The three teacher reported regulation measures of emotional regulation (TRF-DP) (−0.35, 95 % CI [−0.6, −0.2], *p* = 0.001), lability/negativity (ERC-L/N) (−0.48, 95 % CI [−0.9, −0.04], *p* = 0.031), and emotional regulation (ERC-R) (1.73, 95 % CI [1.0, 2.5], *p* ≤0.001) were statistically significant predictors of math scores at second grade. Two of the three teacher-reported regulation measures, the emotional dysregulation (TRF-DP) (−0.28, 95 % CI [−0.5, −0.1], *p* = 0.001) and emotional regulation (ERC-R) (0.78, 95 % CI [0.1, 1.5], *p* = 0.029), were statistically significant predictors of literacy scores at second grade (see Table 5).

## Discussion

The objective of the current study was to explore how early child emotional regulation behaviors were related across informants and settings to better understand state vs trait characteristics of emotional regulation as well as their influence on school adjustment outcomes.

Our first research question examined associations between parental and teacher reports of emotional regulation behaviors in early childhood. Findings revealed inconsistent agreement between parent and teacher reports, with parental reports of lability/negativity most frequently correlating with teacher-reported behavioral dysregulation. Despite inconsistencies, reports within the same informant (e.g., parent-parent or teacher-teacher) showed significant relationships. Prior studies on cross-informant emotional dysregulation measures have shown varied agreement levels, mostly with clinically referred samples, whereas our study utilized a community-based sample (Aitken et al., 2019; Rescorla et al., 2018).

The mixed concordance of emotional regulation behaviors between informants may be indicative of the child's behavioral, emotional, and cognitive regulatory behaviors in different settings (state), i.e. a child may act out more at school than home and thus a teacher may report a lower degree of regulation than a parent (Aitken et al., 2019; De Los Reyes et al., 2015). Parent reports of lability/negativity behaviors which include mood lability, lack of flexibility, dysregulated negative affect, and inappropriate affective displays were more consistently correlated with teacher reports than the regulatory behaviors. Of note, the internal reliability of the ERC Regulation was weak across both parent (0.62) and teacher reports (0.58–0.67), which may reflect how difficult regulatory behaviors are to capture within a self-report measure. This may be because lability/negativity behaviors could be more easily observable than subtle regulatory behaviors (Shields & Cicchetti, 1997). Alternatively, our results may suggest that emotional lability/negativity may be more consistent across settings (trait) compared to regulatory behaviors which may be more dependent on the specific environmental context (state) (Carducci & Nave, 2020). In this case children exhibiting high lability/negativity behaviors can be targeted for intervention before school entry to best support their school adjustment. These differences in trait vs state aspects of emotional regulation behaviors are essential to understand how to best support a child experiencing emotional dysregulation. Alternatively, the inconsistencies between informants may be related to other contextual factors, including parental (parental distress, depressive and anxiety symptoms, income, family functioning), teacher (student-teacher relationship, bias), community (neighborhood, school), and or cultural characteristics (Aitken et al., 2019; Althoff, Rettew, et al., 2010; Collishaw et al., 2009; Rescorla et al., 2018; Roach et al., 2022).

Our second research question was to explore the relationship between parent reported emotional regulation behaviors and teacher reported school adjustment outcomes. Our results were similar to previous studies exploring the relationship between parent reported emotional regulation behaviors and social skills (Dollar et al., 2022; Korucu et al., 2022). Both of the parent reported emotional lability/negativity and regulation measures were significant predictors of social skills that measure a child's self-control, cooperation, responsibility, empathy, and interpersonal skills within the school setting (Elliott et al., 1988; Gould et al., 2011; Gresham & Elliot, 1990). Similarly, our results were consistent with previous studies exploring the relationship between parent reported emotional regulation behaviors and teacher reported academic performance outcomes (Graziano et al., 2007; Li et al., 2017). The parent reported lability/negativity behaviors measure was a statistically significant predictor of all three measures of academic performance. However, the parent reported regulation measure was only significantly associated with academic productivity. Academic productivity is indicative of day-to-day academic performance including how well a child is able to follow directions, complete work in a timely manner, and exhibit independence throughout the day (DuPaul et al., 1991). This contrasts with the academic success and academic competence outcomes that are more indicative of global achievement and accuracy of work completed (DuPaul et al., 1991; Elliott et al., 1988). Our results highlight that parental measures of emotional regulation were associated with a child's ability to adapt to school demands including social skills and academic performance, but not necessarily global academic success measures. However, the parent reported emotional lability/negativity behaviors were consistently related across all three academic adjustment measures indicating that lability/negativity is associated with worse teacher reported social skills, day to day academic functioning, and global academic success. Our results are similar to previous studies, but unique in that we explored both the academic productivity and academic success as separate outcomes, which allows for a more distinct understanding of the relationship of emotional regulation and teacher rated academic performance (Graziano et al., 2007).

Lastly, our final research question was to explore the association between teacher reported emotional regulation behaviors and math and literacy academic achievement scores at second grade. All three teacher reported emotional regulation measures were associated with the standardized achievement math scores, however only the TRF-DP and ERC-R scores were associated with standardized achievement literacy scores. Our results provide further evidence that teacher reports of emotional regulation behaviors are related to academic achievement.

### Clinical applications

Psychiatric mental health nurses can address disparities in emotional dysregulation and school adjustment outcomes as they are uniquely positioned to assist families in navigating socioemotional concerns, including challenges with emotional regulation (McCabe & Best, 2022). Our results reflected that both parent and teacher reports of emotional regulation behaviors were related to school adjustment outcomes. There are several clinical and practice-based implications that can be derived from our work that impact the roles of frontline nurses and advanced practice nurses (across settings including inpatient, outpatient, community, public health, and school nurses), teachers, and caregivers in addressing child psychiatric mental health needs by targeting emotional regulation.

We recommend an interdisciplinary and collaborative approach to screening efforts that includes multiple independent assessments including nurses, caregivers, teachers, psychologists, and/or counselors. This approach to screening allows for the exploration of important trait vs state distinctions for children who are experiencing emotional dysregulation. A recent Pew report indicated mental health is one of parents' major concerns regarding their children (Pew Research Center, 2023). To address this growing concern, emotional regulation may be a key target for nurses in collaboration with parents and other interprofessional school-based stakeholders. Although our study focused on parental and teacher reports of emotional regulation behaviors, primary care, home visiting, and or school nurses have sustained contact with children and their families and play a key role in essential child psychiatric mental health care prevention efforts including screening, referral, and brief interventions (Gross et al., 2016; Jönsson et al., 2019; Molloy et al., 2021; Ravenna & Cleaver, 2016). Galehouse and Foley (2021) provide nurses with a comprehensive resource for integrating emotional regulation principles into psychiatric clinical practice. Nurses can address parental or teacher concerns and stand to reduce access to care barriers, improve family-community continuum of care, and reduce the stigma associated with seeking help (Cowell, 2013). Nurses can work collaboratively with children, families, and school-based stakeholders to disentangle whether emotional dysregulation is related to state or trait characteristics and tailor interventions based on an individual child's needs.

School nurses play a significant role in identifying children's mental health needs with one estimate from Stephan and Connors (2013) indicated that 31 % of health referrals made by school nurses were mental health related. To support these prevention efforts, school-based nurses need to have the necessary supports to address their perceived barriers to care delivery including lack of time, lack of professional confidence, complexity of conditions, lack of training and resources, and large caseloads (Ravenna & Cleaver, 2016). The Mental Health Training Intervention for Health Providers in Schools (MH-TIPS) offers evidencebased resources to enhance competence in addressing student mental health concerns (Bohnenkamp et al., 2019). Nurses that are supported through training, staffing, and system level supports can be empowered to promote positive mental health through individual and school-wide education, screening, early identification, referral to community and professional resources, and brief interventions to promote positive child mental health (Holochwost et al., 2021; Molloy et al., 2021).

The results of our study highlight the importance of incorporating emotional regulation into interventions for children's health and wellbeing. Nurses, psychologists, and teachers should collaborate and implement these interventions across various settings such as parenting, education, early care, home visiting, and school-based programs (Brown et al., 2012; Holochwost et al., 2021; Molloy et al., 2021; Pandey et al., 2018; Waxmonsky et al., 2021). Some examples of existing evidencebased interventions that incorporate developmental psychology and emotional regulation include the Nurse-Family Partnership (NFP) and Early Head Start programs (Dodge, 2015; Nix et al., 2013; Olds et al., 2014).

In our analyses, we found that emotional regulation (ERC-R) had a larger effect size compared to other aspects of emotional dysregulation (ERC-L/N, CBCL-DP, or TRF-DP). Therefore, we recommend a universal approach to promote emotional regulation skills, rather than just targeting children who are experiencing dysregulation (Harrington et al., 2020). Promoting emotional regulation early in life can facilitate positive physical, mental, and school performance outcomes across the lifespan (Blair & Raver, 2015; Hajal & Paley, 2020).

### Limitations/future research

Some limitations need to be considered when interpreting the results of this study. Our analyses used data from 2002 to 2010, which may not be representative of children today. However, few longitudinal studies include developmentally appropriate measures of early childhood emotional regulation across childhood from multiple informants and measure holistic school adjustment outcomes, including social skills, academic performance, and academic achievement. Also, due to the low response rate from teachers completing mail in questionnaires very few children had multiple assessment points for the social skills and academic performance measures. For this reason, we used mean scores to maximize our sample sizes for analysis. However, this did not allow us to explore the longitudinal nature of these relationships and did not account for the potential differences between a child with one, two, or three scores and the potential bias of the differences in teacher reports. Also, we did not account for changes in sociodemographic variables (income and marital status), which may have influenced the relationships of interest. While the majority of measures in this study demonstrated satisfactory internal reliability, a few measures exhibited poor reliability including the ERC-R and the CBCL-DP anxiety/depression subscale. Additionally, the absence of observational or physiological measures of emotional regulation represents a limitation, as these alternatives could mitigate potential biases inherent in parent or teacher reports and improve construct validity (Eberhart et al., 2022). Finally, although this work was guided by the Social Ecological Model, we were only able to account for a limited number of individual and family level characteristics despite additional systemic drivers to the inequities in emotional regulation, academic adjustment, and academic achievement outcomes (Gardner-Neblett et al., 2021; Hoagwood et al., 2018; Peterson et al., 2018). Future research should explore additional factors that contribute to the development of emotional dysregulation behaviors at the individual level (child temperament or genetics), family level (caregiving behaviors and mental health), school (teacher and peer relationships), or societal level (factors associated with low income or structural racism). Despite these limitations, our study was unique in that it simultaneously explored the relationship between developmentally appropriate emotional regulation behaviors and school adjustment outcomes across multiple informants.

## Conclusion

Our study contributes to the literature by providing further evidence of the relationship between emotional regulation and key school adjustment outcomes including social skills, academic performance, and academic achievement. Our findings further indicate the need for multiple informants, including psychiatric nurses, when screening for emotional regulation and highlight that emotional lability/reactivity behaviors may be more consistently identified by teachers and parents compared to regulation behaviors. This information is of great relevance for screening children at risk for emotional dysregulation, and can inform the development of parenting, early care, and education-based interventions to promote optimal health and wellbeing and facilitate children to meet their full social and economic potential across the lifespan.

## Figures and Tables

**Table 1:** Descriptive statistics of sociodemographic variables at 18 months.

Variable	n	% or mean (sd)
Mother age	206	29.3 (5.9)
Mother education	206	-
Less than high school	25	12.1 %
High school or higher	181	87.9 %
Mother marital status	206	-
Married	127	62.6 %
Non-married	79	38.3 %
Income needs ratio	206	-
Above poverty line (>200 %)	100	48.5 %
Below poverty line (≤200 %)	106	51.5 %
Child race/ethnicity	206	-
Black African American	117	56.8 %
White	89	43.2 %
Child assigned sex at birth	206	-
Male	106	51.5 %
Female	100	48.5 %

**Table 2:** Descriptive statistics for emotional regulation measures and academic performance outcomes.

Time (months)	18		24		30		36		60		72		84		Average*	
							
	n	Mean (sd)	n	Mean (sd)	n	Mean (sd)	n	Mean (sd)	n	Mean (sd)	n	Mean (sd)	n	Mean (sd)	n	Mean (sd)
Parent Report CBCL-DP	174	158.0 (11.1)	178	156.3 (9.5)	178	156.4 (9.2)	178	157.5 (12.8)	–	–	–	–	–	–	187	157.2 (8.7)
Parent ERC-Lability/Negativity	–	–	–	–	–	–	–	–	136	27.3 (3.3)	–	–	–	–	–	–
Parent ERC-Regulation	–	–	–	–	–	–	–	–	136	26.2 (5.6)	–	–	–	–	–	–
Teacher Report Form: Dysregulation Profile	–	–	–	–	–	–	–	–	59	160.8 (13.9)	78	161.8 (13.4)	92	162.9 (13.7)	128	163.3 (13.1)
Teacher ERC-Lability/Negativity	–	–	–	–	–	–	–	–	61	22.2 (6.3)	85	22.5 (7.7)	92	22.8 (7.1)	129	23.0 (7.0)
Teacher ERC Regulation	–	–	–	–	–	–	–	–	61	25.1 (3.0)	85	25.3 (4.1)	92	25.2 (3.4)	129	25.1 (3.5)
Teacher APRS Academic Success	–	–	–	–	–	–	–	–	56	27.9 (5.3)	78	26.4 (7.1)	90	26.6 (6.7)	125	26.2 (6.8)
Teacher APRS Academic Productivity	–	–	–	–	–	–	–	–	57	50.6 (8.0)	79	47.0 (10.1)	91	46.5 (9.8)	125	47.0 (9.6)
Teacher SSRS Academic Competence	–	–	–	–	–	–	–	–	61	33.9 (8.3)	86	33.5 (8.8)	91	33.8 (8.5)	129	32.9 (8.5)
Teacher SSRS Social Skills	–	–	–	–	–	–	–	–	61	44.2 (10.5)	86	43.8 (11.1)	91	43.2 (9.9)	129	42.9 (10.2)
WJIII Applied Problems	–	–	–	–	–	–	–	–	–	–	–	–	120	107.5 (16.7)	–	–
WJIII Word Identification	–	–	–	–	–	–	–	–	–	–	–	–	121	108.5 (13.8)	–	–

Note: Emotional Regulation Checklist (ERC), Academic Performance Rating Scale (APRS), Social Skills Rating Scale (SSRS), Woodcock Johnson III (WJII). 60 months = kindergarten, 72 months = first grade, 84 months = second grade. An average score was calculated if a participant had at least one completed measurement across the 60, 72, and 84 month visits.

**Table 3:** Partial correlations of parental and teacher-reported emotional regulation measures.

	CBCL-DP parent	ERC-L/N parent	ERC-R parent	TRF-DP teacher	ERC-L/N teacher	ERC-R teacher
CBCL-DP	x					
ERC-L/N parent	**0.58** **	x				
ERC-R parent	**−0.18** *	**−0.37** **	x			
TRF-DP	0.06	**0.32** *	−0.15	x		
ERC-L/N teacher	0.10	**0.31** *	−0.03	**0.79** **	x	
ERC-R teacher	0.07	−0.18	**0.31** *	**−0.43** **	**−0.44** **	x

Note: CBCL-DP = Child Behavior Checklist Dysregulation Profile (average score 18–36 months), TRF-DP = Teacher Report Form Dysregulation Profile (average score 60, 72, 84 month), ERC-L/N = Emotional regulation Checklist Lability/Negativity (parent = 60 months, teacher = average score of 60, 72, 84 month), ERC-R Emotional Regulation Checklist Regulation (parent = 60 months, teacher = average score of 60, 72, 84 month). Bold = statistically significant * = *p* <.05, ** = *p* < 0.001.

**Table 4:** Effects for parent reported emotional regulation (CBCL-DP, ERC-L/N, ERC-R) on academic adjustment outcomes (social skills (SSRS) and academic performance (APRS-AP, APRS-AS, SSRS-AC).

	CBCL-DP			ERC-L/N			ERC-R		
			
	Effect estimate	95 % CI	p-Value	Effect estimate	95 % CI	p-Value	Effect estimate	95 % CI	p-Value
*Social skills: SSRS-SS*									
Emotional regulation	0.1	(−0.2, 0.3)	0.513	**−0.4**	**(−0.6, −0.1)**	**0.022**	**0.57**	**(0.02, 1.12)**	**0.042**
Sex assigned at birth	**5.8**	**(2.4, 9.2)**	**0.001**	**5.4**	**(2.0, 8.8)**	**0.002**	**5.44**	**(2.05, 8.83)**	**0.002**
Race/ethnicity	1.6	(−2.0,5.2)	0.371	2.3	(−1.3, 5.9)	0.201	2.14	(−1.46, 5.74)	0.241
Maternal education	−0.6	(−6.0, 4.7)	0.816	0.6	(−4.8, 5.9)	0.838	−0.66	(−6.03, 4.70)	0.807
Marital status	3.2	(−3.3, 9.6)	0.335	−0.5	(−7.4, 6.4)	0.890	1.08	(−5.91, 8.07)	0.759
Income	**−6.1**	**(−11.1, −1.1)**	**0.018**	−3.5	(−8.8, 1.7)	0.188	−3.09	(−8.47, 2.29)	0.257
*Academic performance: APRS-AP*									
Emotional regulation	−0.01	(−0.2, 0.2)	0.927	**−0.5**	**(−0.7, −0.2)**	**0.002**	**0.75**	**(0.2, 1.3)**	**0.005**
Sex assigned at birth	2.2	(−1.1, 5.6)	0.181	2.1	(−1.1, 5.4)	0.193	2.19	(−1.1, 5.5)	0.189
Race/ethnicity	1.7	(−1.7, 5.2)	0.324	1.3	(−2.2, 4.7)	0.467	1.02	(−2.5, 4.5)	0.562
Maternal education	−2.2	(−7.4, 3.1)	0.417	−0.1	(−5.4, 5.1)	0.964	−1.77	(−7.1, 3.6)	0.511
Marital status	−2.4	(−8.5, 3.8)	0.449	−6.1	(−12.6, 0.4)	0.067	−4.04	(−10.7, 2.6	0.229
Income	−4.2	(−9.1, 0.7)	0.092	−3.3	(−8.4, 1.9)	0.215	−2.63	(−8.0, 2.7)	0.331
*Academic performance: APRS-AS*									
Emotional regulation	0.00	(−0.2, 0.2)	0.957	**−0.3**	**(−0.5, −0.1)**	**0.003**	0.30	(−0.1, 0.7)	0.117
Sex assigned at birth	0.49	(−1.8, 2.8)	0.671	0.5	(−1.8, 2.7)	0.678	0.53	(−1.8, 2.9)	0.652
Race/ethnicity	1.30	(−1.1, 3.7)	0.288	1.1	(−1.3, 3.5)	0.361	1.03	(−1.5, 3.5)	0.413
Maternal education	−1.50	(−5.1, 2.1)	0.413	−0.1	(−3.7, 3.6)	0.973	−0.93	(−4.7, 2.9)	0.627
Marital status	−3.01	(−7.2, 1.2)	0.162	**−4.6**	**(−9.1, <0.01)**	**0.049**	−3.53	(−8.3, 1.2)	0.141
Income	−3.28	(−6.7, 0.1)	0.058	−2.8	(−6.4, 0.8)	0.131	−2.84	(−6.6, 1.0)	0.143
*Academic performance: SSRS-AC*									
Emotional regulation	0.04	(−0.1, 0.2)	0.654	**−0.4**	**(−0.6, −0.2)**	**0.001**	0.26	(−0.2, 0.7)	0.268
Sex assigned at birth	1.12	(−1.7, 3.9)	0.426	1.1	(−1.6, 3.8)	0.439	1.14	(−1.7, 4.0)	0.427
Race/ethnicity	2.43	(−0.5, 5.4)	0.104	2.2	(−0.7, 5.0)	0.137	2.12	(−0.9, 5.1)	0.163
Maternal education	−0.80	(−5.1, 3.5)	0.715	0.7	(−3.5, 5.0)	0.735	−0.23	(−4.7, 4.2)	0.919
Marital status	−5.04	(−10.3, 0.2)	0.059	**−7.4**	**(−13.0, −1.9)**	**0.009**	**−6.34**	**(−12.2, −0.5)**	**0.033**
Income	**−4.85**	**(−8.9, −0.8)**	**0.020**	−3.4	(−7.6, 0.9)	0.117	−3.74	(−8.2, 0.8)	0.101

Note: CBCL-DP: Child Behavior Checklist-Dysregulation Profile, ERC-L/N: Emotional regulation Checklist-Lability/Negativity, ERC-R: Emotional Regulation Checklist-Regulation, SSRS-SS: Social Skills Rating System-Social Skills, APRS-AP: Academic Performance Rating Scale-Academic Productivity, APRS-AS: Academic Performance Rating Scale-Academic Success, SSRS-AC: Social Skills Rating System-Academic Competence. Bold: Statistically significant *p* < 0.05.

**Table 5:** Effects for teacher reported emotional regulation (TRF-DP, ERC-L/N, ERC-R) on academic achievement outcomes (WJIII-AP & WJIII-LWI) at second grade.

	TRF-DP			ERC-L/N			ERC-R		
		
	Effect estimate	95 % CI	*p*	Effect estimate	95 % CI	*p*	Effect estimate	95 % CI	*p*
*Standardized assessment: WJIII-AP*									
Emotional regulation	**−0.35**	**(−0.6, −0.2)**	**0.001**	**−0.48**	**(−0.9, −0.04)**	**0.031**	**1.73**	**(1.0, 2.5)**	**<0.001**
Sex assigned at birth	−1.05	(−6.2, 4.1)	0.684	−2.87	(−8.4, 2.7)	0.310	−4.80	(−10.1, 0.5)	0.076
Race/ethnicity	**11.82**	**(6.5, 17.2)**	**<0.001**	**12.79**	**(7.2, 18.4)**	**<0.001**	**12.84**	**(7.6, 18.1)**	**<0.001**
Maternal education	−1.02	(−9.0, 7.0)	0.800	0.59	(−7.9, 9.1)	0.892	−3.56	(−11.6, 4.5)	0.381
Marital status	−1.31	(−12.2, 9.6)	0.812	−2.99	(−14.6, 8.6)	0.610	−5.96	(−16.9, 5.0)	0.284
Income	**−8.53**	**(−16.3, −0.8)**	**0.032**	**−9.24**	**(−17.4, −1.1)**	**0.027**	−4.88	(−12.9, 3.1)	0.227
*Standardized assessment: WJIII-LWI*									
Emotional regulation	**−0.28**	**(−0.5, −0.1)**	**0.001**	−0.18	(−0.6, 0.2)	0.330	**0.78**	**(0.1, 1.5)**	**0.029**
Sex assigned at birth	0.12	(−4.3, 4.5)	0.956	−0.47	(−5.2, 4.2)	0.843	−1.44	(−6.1, 3.2)	0.544
Race/ethnicity	2.61	(−2.0, 7.2)	0.262	2.96	(−1.8, 7.7)	0.218	2.97	(−1.7, 7.6)	0.208
Maternal education	−2.96	(−9.8, 3.9)	0.394	−2.70	(−9.9, 4.5)	0.460	−4.46	(−11.5, 2.6)	0.215
Marital status	**−14.11**	**(−23.4, −4.8)**	**0.003**	**−14.69**	**(−24.5, −4.9)**	**0.004**	**−16.10**	**(−25.8, −6.4)**	**0.001**
Income	−2.92	(−9.6, 3.8)	0.387	−4.03	(−10.9, 2.9)	0.250	−2.04	(−9.1, 5.0)	0.566

Note: TRF-DP: Teacher Report Form-Dysregulation profile, ERC-L/N: Emotional regulation Checklist-Lability/Negativity, ERC-R: Emotional Regulation Checklist: Regulation, WJIII: Woodcock Johnson III, bold: Statistically significant p < 0.05.
